# The Complex Dynamics of Resources and Maintaining Factors in Social Networks for Alcohol-Use Disorders: A Cross-Sectional Study

**DOI:** 10.3389/fpsyg.2022.804567

**Published:** 2022-03-17

**Authors:** Niels Braus, Sonja Kewitz, Christina Hunger-Schoppe

**Affiliations:** ^1^Department of Psychology and Psychotherapy, Witten/Herdecke University, Witten, Germany; ^2^Department of Psychotherapy for Children and Adolescents, Institute for Psychology, Goethe University Frankfurt, Frankfurt, Germany

**Keywords:** social network, alcohol use disorder, social support, social negativity, craving, Social Network Interview, systemic therapy

## Abstract

Systemic therapy considers the complex dynamics of relational factors and resources contributing to psychological symptoms. Negative maintaining factors have been well researched for people suffering from Alcohol-use Disorders (AUD). However, we know little about the complex dynamics of these negative factors and resources. We interviewed fifty-five participants suffering or fully remitted from Alcohol-use disorders in this cross-sectional study (*M* = 52 years; 33% female). The interviews focused on relational factors (e.g., social support and social negativity) referring to a Support Social Network and a Craving Social Network (CSN). The CSN included all significant others who were associated with craving situations. We compared the network characteristics of the group suffering from Alcohol-use Disorders (*n* = 38) to a fully remitted control group (*n* = 17). The abstinent group with full remission named on average fewer individuals in the CSNs. They had lower social negativity mean scores in the Support Social Network compared to the non-remitted group (*d* = 0.74). In the CSN, the mean scores of social support were significantly higher than the median for both groups (*d* = 2.50). These findings reveal the complex interplay of relational patterns contributing to the etiology, maintenance, and recovery from Alcohol-use disorders. A successful recovery can be linked to increased social resources and reduced relations associated with craving. However, craving-associated relations represent an important source of social support. Future research should investigate this ambivalence for the systemic perspective on the explanation and treatment of Alcohol-use disorders.

## Introduction

Systemic therapy is a widespread evidence-based psychotherapy approach. According to the systemic perspective, mental disorders are understood within the context of social systems or networks (von Sydow et al., 2010). Therefore, significant others are included directly or virtually in the therapy (e.g., systems-oriented questioning and genogram work) (Becvar and Becvar, 2009).

Apart from this theoretical view, recent empirical studies investigating psychological health in general (Hartmann et al., 2010) showed that an integration of significant others within therapy is beneficial for both the client and the significant others. Therefore, research on the development of diagnostic instruments assessing social networks has been stimulated. Complex measures show more predictive validity in assessing social networks than simple measures do (Holt-Lunstad et al., 2010, 2015). Related research to Alcohol-use disorders (AUD; Litt et al., 2016) asserts that larger, integrated social networks can be more easily activated to protect individuals from drinking than disjointed social networks (Perry and Pescosolido, 2012; Pescosolido, 2015).

Systemic therapy historically focused on family systems (von Sydow et al., 2010). The modern approach of open dialogue also draws on systemic theory. It exceeds the historical focus on family systems highlighting the role of social networks including extra-familial and professional relations (Breunlin et al., 2011; Heatherington et al., 2015). Therefore, this approach uses network meetings as a central intervention (Seikkula et al., 2001; Razzaque and Stockmann, 2016; von Peter et al., 2019). However, due to a variety of other specificities, open dialogue is not considered as a systemic approach. Yet, it highlights the importance of including social networks around an individual in psychotherapy based on a systemic theory. Likewise, the important role of social networks for recovery has also been recognized in the research field of alcohol and drug use disorders (Mericle, 2014; Stone et al., 2016). Most studies use the Important People Interview (IPI) (Zywiak et al., 2009) to assess social network characteristics. The IPI and other validated methods of social network instruments have discovered central variables to predict important alcohol use disorder-specific outcomes (Homish and Leonard, 2008; Longabaugh et al., 2010; Majer et al., 2015). A growing body of research (Goehl et al., 1993; Howard, 2006; Young and Timko, 2015) highlights the interpersonal dilemmas associated to positive social drifts in course of recovery. Identifying and supporting individuals in retaining and abandoning certain network members seem to be essential to treatment success (Goehl et al., 1993; Stone et al., 2014; Young and Timko, 2015). In her review on social networks with AUD, Mericle (2014) demands more future research discovering the complex dynamics within social networks in order to develop specific network treatments and strategies. With regard to the development of therapeutic practice, Mericle (2014) demands more knowledge on temporal processes, resources and barriers that help therapists understand how to support individuals facing social drifts during the recovery process.

In the course of an intervention study on systemic therapy for social anxiety (Hunger et al., 2020), a new social network instrument was developed. The so-called Social Network interview (SocNet) aimed for assessing the complex interpersonal processes during treatment. The SocNet is a semi-structured instrument based on hierarchical mapping technique by Antonucci (1986). It integrates both qualitative as well as structural social network characteristics in line with the recommendation by Mericle (2014). The study at hand transfers the SocNet to the context of AUD. However, one could question the need for a more complex instrument given the valid and simple IPI. We on the other hand believe that the SocNet instrument could inspire and support future intervention studies considering the complexities of real-life social networks. The MATCH-project (Longabaugh et al., 2010) represents an example for the development of treatment approaches based on findings on social networks. The authors discovered the importance of network structure and support for treatment success. Litt et al., 2016 then utilized the findings of the MATCH-project to develop an individually delivered social network intervention. This treatment supports individuals suffering from AUD to alternate their social networks according to findings from the MATCH-project. In the study on systemic therapy (Hunger et al., 2020), the SocNet has shown to support therapists in integrating relevant social network members into therapy. We believe that the SocNet could also inspire novel network interventions that are not individually delivered but are delivered in a similar way to the network meetings of open dialogue (Seikkula et al., 2001).

In the SocNet (Hunger et al., 2019) a resource-oriented support social network (SSN) was added to the assessment of a problem-orientated social network. This additional perspective exceeds the IPI. The resource-oriented support network encompasses all important others who support the participant to deal with everyday life. In order to transfer the problem-orientated social network to the context of this study, it now refers to a craving social network (CSN). The CSN encompasses all important others who are associated to or are a trigger to craving. According to the DSM, craving is “a strong desire or urge to use alcohol” (American Psychiatric Association, 2013). Alcohol craving is a central variable in the theoretical models regarding the etiology, maintenance and recovery of AUD (Schlauch et al., 2019). More recent models and findings based on social learning theory have stressed the importance of social situations and cues in the context of craving (Marlatt, 1996; Drummond et al., 2000).

In order to receive even more insights into the dilemmatic dynamics, social support encompasses both positive social support and social negativity. Several studies revealed that these qualitative network characteristics have more predictive power for the recovery of AUD (Longabaugh et al., 2010; Mericle, 2014) than structural aspects of social networks. This concept of social support is based on social exchange theory (Cook et al., 2013) differentiating between positive and negative socio-emotional exchanges. Positive social support describes positive socio-emotional exchanges (e.g., acceptance, altruistic behavior) (Bertera, 2005). Social negativity describes a negative form of socio-emotional exchange (e.g., lack of empathy and acceptance or debasement) or can encompass the subjective conflicting aspect of a relationship (Bertera, 2005). In this study we assume that both forms of social support are present within the same social network (i.e., within the SSN and CSN). Moreover, including social negativity was not only based on theoretical concerns but evidence-based: While findings on positive social support in the prediction of patients’ health are inconsistent (Stone et al., 2016), a growing body of research confirms the predictive advantage of social negativity for patients’ health outcomes (Schuster et al., 1990). Although positive social support seems to be relevant for the recovery of AUD, social support showed less predictive power for alcohol-related outcomes than social negativity (Beattie and Longabaugh, 1999). Various findings of longitudinal studies (Kelly et al., 2011; McCutcheon et al., 2014; Brown et al., 2015) indicate patterns of social drifts of successfully recovered persons with AUD: Successfully recovered patients report that the number of network members associated to drinking decreases and the amount of positive social support increases.

## Hypotheses and Research Question

### Differences Between Craving- and Support Social Networks

*Hypothesis 1a* We assume that the degree of positive social support within the support social network is significantly higher compared to the CSN. We also assume that the degree of social negativity within the CSN is significantly higher compared to the support social network.
*Hypothesis 1b* We assume that the degree of social negativity in social support networks is significantly higher than the minimum of the scale. We also assume that the degree of positive social support in craving support networks is significantly higher than the minimum of the scale.

*Research Question 1:* What are the differences between craving- and support social networks concerning other structural aspects?

### Differences Between Remitted vs. Non-remitted Participants

*Hypothesis 2* We assume that the individuals recovered from AUD name more people within support social networks and fewer within craving networks. We assume that the persons recovered from AUD experience more positive social support and experience less social negativity in both networks.

*Research Question 2:* What differences can be found concerning other characteristics within the craving- and support social networks (CSN, SSN) between persons recovered from AUD and suffering from AUD?

## Methods

### Design

This study is a monocentric cross-sectional pilot trial. The sample consists of patients who currently suffer or have suffered from an alcohol use disorder in terms of dependency (F10.2) or abuse (F10.1) (Dilling et al., 2014). The Ethics Committee of the Medical Faculty of Heidelberg University (S-524/2018) approved the project.

### Participants

Participants had to fulfill the following inclusion criteria: (1) age > 18 years; (2) diagnosis of an alcohol use disorder in terms of dependency or abuse (ICD-10: F10.1, F10.2); (3) informed consent. Comorbid disorders were allowed as long as the AUD was of primary concern. Interested persons with the following criteria were excluded: (1) Diseases accompanied with a severe impairment of neurological or cognitive functioning; (2) acute intoxication at the time of the interview; (3) eating disorder (BMI < 14); (4) acute psychotic disorder (F23.x); (5) insufficient German language skills. Recruitment was performed by distributing flyers and study information to physicians, psychologists, psychotherapists, occupational therapists, hospitals and psychosocial counseling centers in and around Heidelberg, public announcements in the local press, on the website of the Institute for Medical Psychology at the University Hospital Heidelberg, and Facebook. All participants presented themselves and none were referred.

### Procedure

In order to participate, each interested person had to participate in a screening interview addressing the persons’ drinking behavior (SCID, section E for alcohol use disorder; Wittchen et al., 1995), additional psychological and medical problems, previous and current psychotherapy and/or pharmacotherapy, self-harm as well as the tendency to injure others, and suicidality. Either the persons’ general practitioner or one of the psychologists from our study team performed the semi-structural screening interview. If the person met the inclusion criteria, she or he was invited to the SocNet. Prior to this, the person was asked to fill out a questionnaire with standardized scales on drinking behavior and mental health.

The SocNet started with the support social network followed by the CSN. After having finished the SocNet, the participants received a photo from their support and CSN if desired. All participants were invited to call the interviewer and/or the study team in case of any discomfort subsequent to the interview session.

### Material

#### The Social Network Interview

The SocNet is a semi-structured interview, assessing two types of social networks: support social networks (SSN) and CSN. SSN include (groups of) individuals which support a person to cope with everyday life situations in a confident and secure manner. Participants are shown a diagram depicting three concentric circles (Supplementary Material 1). The center of the smallest circle displays the word “I.” Participants were asked to think about (groups of) people of whom they perceived very much (circle 1), some (circle 2), or a bit social support (circle 3). There was an additional category of (groups of) people who showed no social support though they wished to be supported by them (circle 4). Participants were asked to place wooden blocks into the circles representing the (groups of) individuals based on how much the participants felt supported by them. Participants were then asked detailed questions about the (groups of) individuals. For individuals, questions included age of each person, kind and duration of the relationship, and frequency of contact. For groups of individuals, patients estimated mean age and range of age, the number of individuals constituting the group, duration of the relationship, and frequency of contact to the group, in addition to the description of the kind of relationship with this group. Considering the three (groups of) most important individuals within the social network, questions also referred to perceived positive social support and social negativity.

CSN include (groups of) individuals who stimulated craving and/or represented situations in which the participants experienced craving when coping with everyday life situations. We adapted the above described SSN depicting three concentric circles while, again, the center of the smallest circle displayed the word “I” (Supplementary Material 1). Participants were asked to think about (groups of) people who stimulated no craving (circle 1), some (circle 2) or much craving (circle 3), or no craving though supposed to stimulate it (circle 4), and to place wooden blocks into the circles representing the (groups of) individuals based on how much they caused craving perceived by the participants. Participants were then asked detailed questions about the (groups of) individuals including age of each person, kind and duration of the relationship, and frequency of contact. Considering the three (groups of) individuals by whom the participants felt most craving, questions again referred to perceived positive social support and social negativity.

In accordance to Bertera (2005) and Sherman et al. (2013), we assessed positive social support and social negativity associated with the three (groups of) individuals by whom the participants felt most support or craving for the SSN and CSN, respectively (for the items see Supplementary Material 2). All items were rated on a four-point rating-scale from 1 (not at all) to 4 (very much). In our study, Cronbach’s alpha for positive social support in the SSN was at 0.77 and in the CSN at 0.92. Cronbach’s alpha for social negativity in the SSN was at 0.76 and in the CSN at 0.93.

#### Validated Instruments for Drinking Behavior

The German version of the Alcohol Abstinence Self-Efficacy scale (AASE, DiClemente et al., 1994; German-version: KAZ-35: Körkel and Schindler, 1996; Schindler et al., 1997) assessed the self- efficacy to stay abstinent in potential relapse situations.

The motivation to change was assessed by the German short version of the University of Rhode Island Change Assessment Scale (URICA, Mander et al., 2012). This instrument was adopted for the purposes of this study changing the open conceptualization of the problem to “problems related to drinking behavior.”

### Statistical Analysis

The statistical analyses were calculated using SPSS, Version 25.0 (IBM, Germany). The calculation of the structural aspects (i.e., size, age, composition, sustainability, frequency of contact) encompassed those people who were placed within the three concentric circles. The overall network size represented the total number of persons constituting the SSN or CSN, respectively. We calculated the mean values and standard deviations for all additional structural aspects (i.e., age, composition, sustainability, frequency of contact). We also identified these descriptive data for the functional aspects (i.e., positive social support and social negativity) including the three most important significant others.

Depending on the distribution of the variables, a paired *t*-test or a Wilcoxon signed rank-test served to determine any differences in structural or functional aspects between both social networks (RQ1/H1a). To test Hypothesis 1b, a one-sample *t*-test compared the sample’s mean against the test value of the arithmetical minimum and the median was conducted. Analogously to research question 1, depending on the distribution, an unpaired two-sample *t*-test or Mann-Whitney-*U*-Test was used to compare the network characteristics of remitted vs. non-remitted participants (RQ2/H2).

## Results

### Sample

The sample consisted of 55 participants, a third (33%) of them were female and more than a half of them (51%) lived in a partnership or marriage. The average age was 52 years (*SD* = 16.71), with almost half of the sample being retired (44%). About a third (31%) of the participants stated to have achieved a complete remission of AUD. The participants with a completed remission had significant higher abstinence self-efficacy [*U*(1, 55) = 85.50, *p* < 0.001, *d* = 1.17] but there was no significant difference in the Committed Action indicator for motivation to change. The majority of participants fulfilled the criteria of an alcohol dependency syndrome (84%) while the diagnosis of alcohol harmful use (16%) characterized a minority (Supplementary Material 3).

### Differences Between Craving- and Support Social Networks

H1a: Referring to hypothesis 1a, participants received more positive social support from members in the SSN compared to the CSN [*t*(37) = 6.00; *p* < 0.001, *d* = 0.97] and more social negativity from the members in the CSN compared to the SSN, [*t*(37) = –4.87; *p* < 0.001, *d* = 0.79 (Table 1)].

**TABLE 1:** Structural and qualitative aspects compared between the Support and the Craving Social Networks.

	Support social networks			Craving social networks					
	*n*	*M*	*SD*	*n*	*M*	*SD*	*t (n)*	*p*	*d*
*Social support*	55	3.41	0.35	38	2.77	0.71	6.00 (37)	<0.001	0.97
*Social negativity*	55	1.68	0.37	38	2.21	0.72	–4.87 (37)	<0.001	–0.79
*Sustainability* (month)	55	264.98	136.55	40	291.97	241.13	–1.61 (39)	0.116	–0.254
*Frequency of contact*^a^**	55	3.19	0.98	40	2.62	1.38	1.86 (39)	0.071	0.294

	***n***	***M***	***SD***	***n***	***M***	***SD***	***z (n)***	***p***	***r***

*Network size*	55	29.64	75.00	40	303.40	450.23	–1.67 (40)	0.100	0.264
*Age* (years)*^b^*	55	48.63	10.73	39	47.88	16.42	–0.32 (39)	0.747	0.050

*Annotation. All values of groups are relativized by group size.*

*^a^Scale from 1 to 8 (1 = daily; 2 = more than once a week; 3 = once a week; 4 = more than once a month; 5 = once a month; 6 = more than once a year; 7 = once a year; 8 = less frequent).*

*^b^Age means average age of network members.*

H1b: The social negativity in the SSN [*t*(55) = 6.00; *p* < 0.001, *d* = 2.50] and the positive social support in the CSN [*t*(37) = 13.72; *p* < 0.001, *d* = 1.85] were significantly higher than the arithmetical minimum. The positive social support in the CSN was even significantly higher than the median of the scale [*t*(37) = 2.36; *p* < 0.05, *d* = 2.50], while social negativity in the SSN was significantly lower than the median of the scale, [*t*(55) = –16.55; *p* < 0.001, *d* = –2.23].

RQ 1: There was no significant difference considering structural aspects (i.e., size; age; composition: private persons, work-related persons; innovation; quantity) between the CSN and SSN, with the only exception of professionals to the advantage of the SSN, *z* = –3.45, *p* < 0.001, *r* = –0.556, and others to the advantage of the CSN, *z* = –2.67, *p* < 0.0081, *r* = –0.41 (Table 1).

### Differences Between Remitted vs. Non-remitted Participants

*H2:* More than half of the remitted participants (53%) reported to have no CSN while only 16% of the participants who are not completely remitted reported no CSN [χ^2^(1, 40) = 8.17, *p* < 0.01, *V* = 0.03]. Participants with complete remission had significantly smaller craving networks compared to non-remitted participants, *U* = 58.00, *z* = –2.39, *p* < 0.05, *r* = –0.38. There was no difference in the size of SSN, *U* = 32.00, *z* = 0.00. With regard to social support and social negativity, there was only one significant difference: Remitted participants had lower rates of social negativity in the support social network than non-remitted [*t*(53) = 2.52, *p* < 0.05, *d* = 0.74].

*RQ 2:* Similarly to the age of participants with remission, their important others in the SSN were significantly older compared to the non-remitted group (*U* = 145.00, *z* = –3.24, *p* = 0.001, *r* = –0.44) and they have known each other for a longer period of time, U = 197.00, *z* = –2.30, *p* < 0.05, *r* = –0.31. The remitted participants reported to have less contact to their important others in the CSN, *U* = 45.50, *z* = –2.82, *p* < 0.01, *r* = –0.45. There were no significant differences in the other SSN and CSN network characteristics between the remitted and non-remitted group (Supplementary Material 4).

## Discussion

Given the limited sample size of this pilot, the results should be interpreted with caution. Nevertheless, the findings indicate how the application of SocNet could help clients, practitioners and researchers to discover the ambiguity of social relations with regard to AUD.

As predicted (hypothesis 1a), the SSN is characterized by a higher degree of positive social support and the CSN by a higher degree of social negativity. However, as supposed by hypothesis 1b, you can find some degree of social negativity in the SSN and even a medium degree of positive social support in the CSN. Referring to the differences between participants with and without remission (hypothesis and research question 2), participants with remission report more frequently to have no CSN and to have smaller CSN as well as to have less social negativity in their SSN.

These findings show once more the complexity of social relationships and its dynamics in the context of AUD (Mericle, 2014). In current research on the recovery of AUD (Mericle, 2014; Litt et al., 2018), patients are suggested to quit their CSN and to foster relationships with people outside of it. However, the findings of our study show the ambivalence of these social drifts. The relationships in the CSN seem to be associated with a medium degree of social support. Therefore, the findings show that through quitting the CSN individuals also could lose social support. Nonetheless, people remitted from AUD show smaller or no CSN in comparison to people with non-remitted AUD. This also reveals the systemic interrelations explaining AUD introduced by a social network perspective (Becvar and Becvar, 2009).

Thus, the current findings also imply that helping professionals could support concerned individuals in finding ways to keep less in touch or even quit their CSN through fostering their SSN. Therefore, the findings show the importance of incorporating social relationships into psychotherapy of AUD (von Sydow et al., 2010; Breunlin et al., 2011; Carr, 2019). To support individuals in the recovery process, these findings could also suggest the implementation of systemic social network approaches (Seikkula et al., 2001; Breunlin et al., 2011; von Peter et al., 2019) for the treatment of AUD.

### Strength

This pilot trial suggests a more comprehensive approach for the study of social network characteristics of persons with AUD, through using the SocNet. This instrument could be helpful for future research in order to understand complex social structures behind any psychological disorders. In this context, the study at hand replicates the findings of a previous study on the SocNet in the context of social anxiety (Hunger et al., 2019) confirming the SocNet as an effective instrument to gain more insights into the ambiguity and complexity of social networks. Especially the medium degree of positive social support in the CSN adds to the line of research supposing a dilemmatic nature of relationships with regard to AUD (Goehl et al., 1993; Howard, 2006; Young and Timko, 2015).

The cross-sectional differences between participants with remission vs. non-remission replicate the previous longitudinal findings on a successful recovery of AUD (Kelly et al., 2011; McCutcheon et al., 2014; Brown et al., 2015). Nevertheless, the introduction of a distinction between SSN and CSN extends these findings and indicates that some successfully remitted participants have maintained some contacts to important others associated to drinking.

### Limitations

While this study is a pilot trial with limited sample size, this publication follows current research ethics that claim the significance of discussing such kind of studies before the realization of a fully powered research project (Arain et al., 2010; Thabane et al., 2010; Leon et al., 2011).

Additionally, it can be enhanced by the inclusion of external or behavioral criteria such as the common TFLB (Dulin et al., 2017) added by biological markers (e.g., heart rate variability, skin conductance response) and while controlling for social desirability. As several remitted participants gave us feedback that they have not understood the proper meaning of the motivation to change scale in their situation, future research could integrate other scales of motivation and check for this concern (Heidenreich and Hoyer, 2001).

Compared to comprehensive research studies, a major limitation of this study is its cross-sectional design. A longitudinal design within an intervention study could have several advantages: Firstly, assessing social networks before and after psychotherapy allows for a more precise evaluation of the SocNet’s validity. Secondly, a longitudinal design adds to the external validity with regard to the development of social network interventions. Thirdly, a longitudinal design would probably lead to a higher number of CSN and it would be easier to compare group sizes because all participants would start as non-remitted.

## Data Availability Statement

The datasets presented in this article are not readily available because raw data cannot be anonymized. Requests to access the datasets should be directed to NB, niels.braus@uni-wh.de.

## Ethics Statement

The studies involving human participants were reviewed and approved by the Ethics Committee of the Medical Faculty of Heidelberg University. The patients/participants provided their written informed consent to participate in this study.

## Author Contributions

NB drafted the brief research report. CH-S and SK helped edit the manuscript and approved the final manuscript. All authors contributed to the design of the manuscript.

## Conflict of Interest

The authors declare that the research was conducted in the absence of any commercial or financial relationships that could be construed as a potential conflict of interest.

## Publisher’s Note

All claims expressed in this article are solely those of the authors and do not necessarily represent those of their affiliated organizations, or those of the publisher, the editors and the reviewers. Any product that may be evaluated in this article, or claim that may be made by its manufacturer, is not guaranteed or endorsed by the publisher.

## Abbreviations

AUD: alcohol use disorder
ICD: International Classification of Diseases
SocNet: Social Network Interview
SSN: Support Social Network
CSN: Craving Social Network
H1: Hypothesis 1
RQ: Research Question.
