# Supplementary material for: The Complex Dynamics of Resources and Maintaining Factors in Social Networks for Alcohol-Use Disorders: A Cross-Sectional Study
Source: Front Psychol. 2022 Mar 17;13:804567. doi: 10.3389/fpsyg.2022.804567 (PMC8968857; doi:10.3389/fpsyg.2022.804567)
Supplement: Supplementary file 1 [file Data_Sheet_1.pdf]

Supplementary Material

1 Supplementary Figures

1.1 Supplementary Material S1

1.1.1 Support Social Network

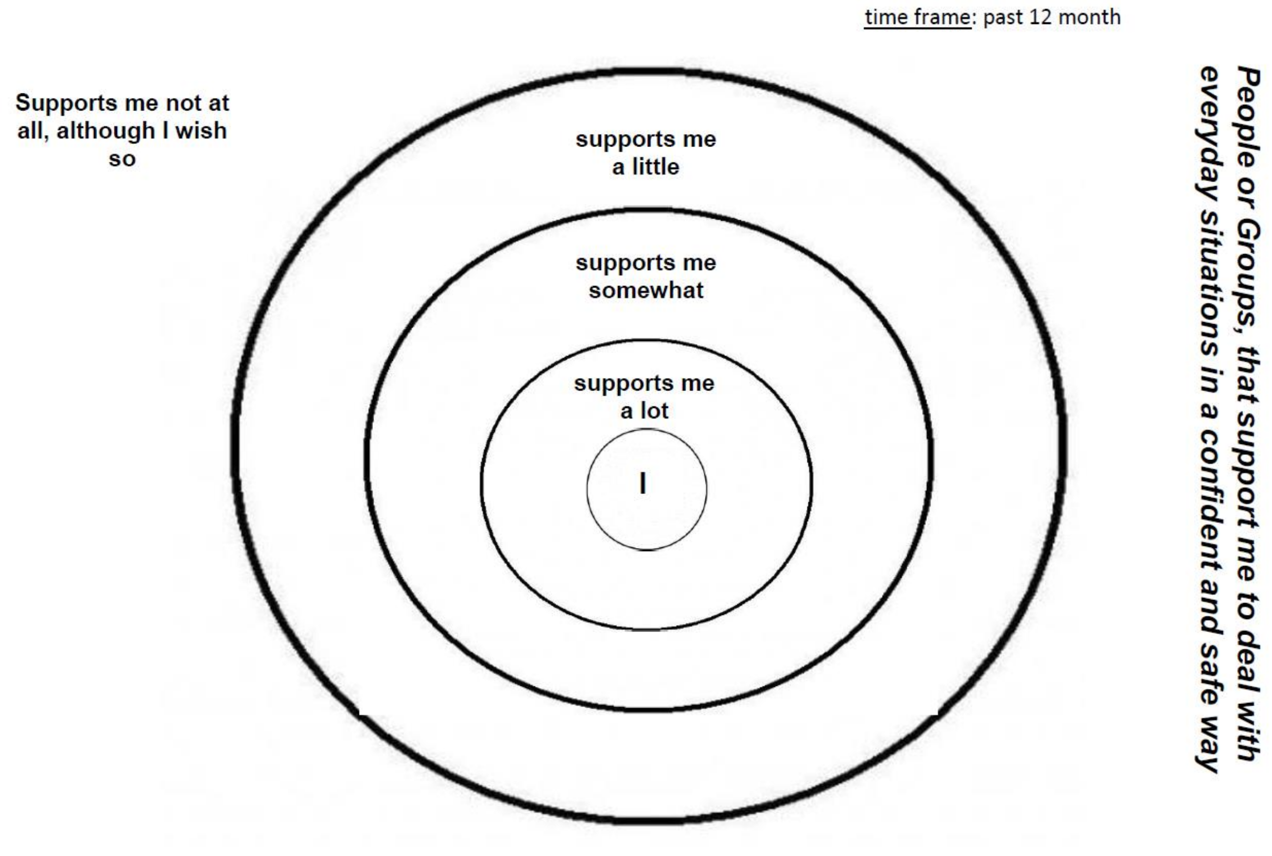

Figure 1: Illustration of the circles in the Craving Social Network.

1.1.2 Craving Social Network

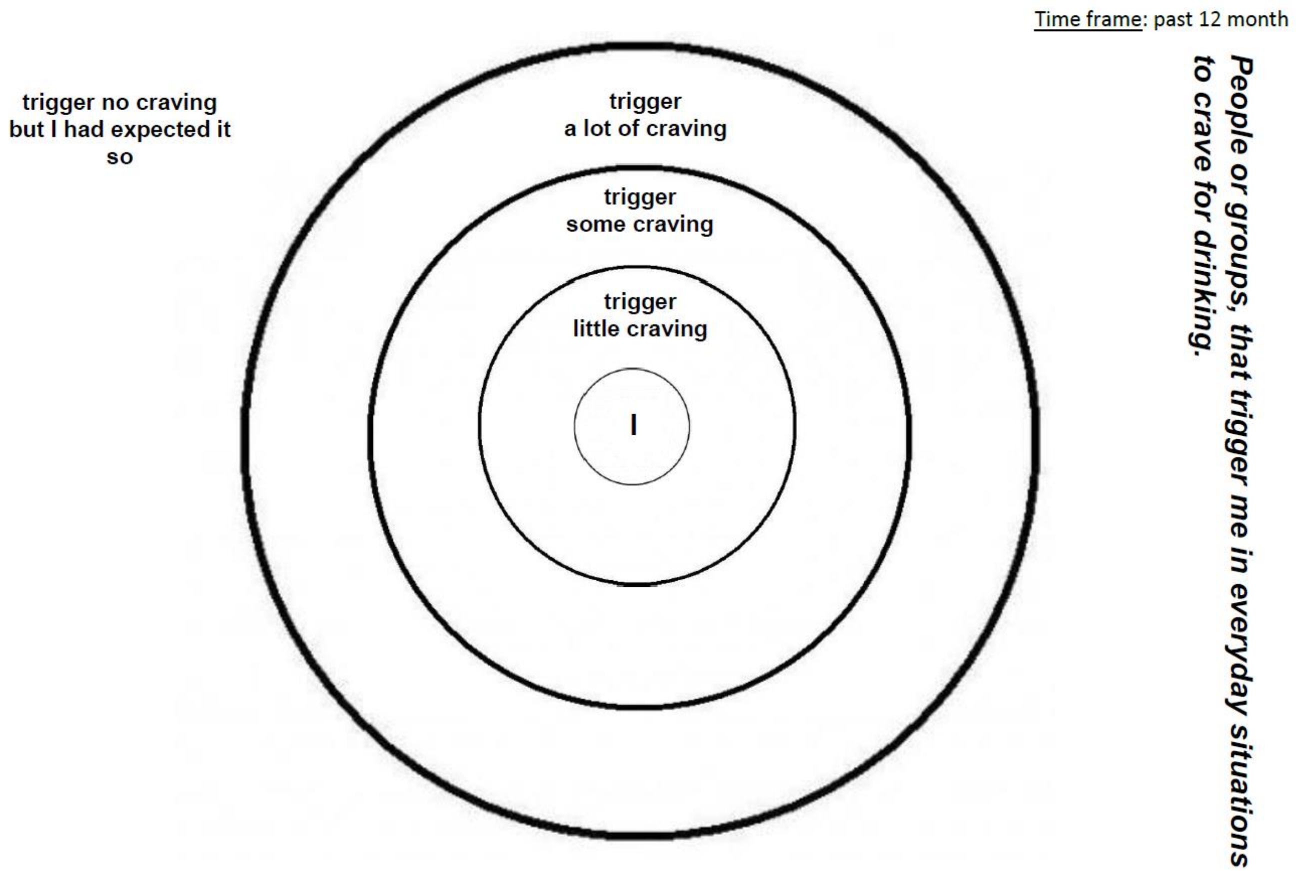

Figure 2: Illustration of the circles in the Craving Social Network.

2 Supplementary Tables

2.1 Supplementary Tables S2

Table 1

Items on positive social support and social negaticity..

**Positive social support**	**Social negativity**
(1) How much do you feel being cared by [person's name]?";	(1) How much do you feel that [person's name] demands to much from you?";;; (5)
(2) How much do you feel being understood by [person's name]?	(2) How much does [person's name] gets on your nerves?"
(3) How much do you feel that you can call upon [person's name] when having problems?";	(3) How much do you argue with [person's name]?";
(4) How much do you feel being supported by [person's name]?	(4) How much do you feel criticized by [person's name]?
(5) "How much do you feel that [person's name] sticks to you?";	(5) How much do you feel that [person's name] lets you down?";
(6) "How much does [person's name] motivates you to take action?".	(6) How much does [person's name] hinder you to take action?".

2.2 Supplementary Tables S3

Table 2

Sample Description.

	Total (n = 55)		Remission (n = 17)		No Remission (n = 38)		Between-Group Differences[a]			
	M	*SD*	*M*	*SD*	*M*	*SD*	*U*		*p*	*d*
Age (years)	51.82	16.17	61.59	12.23	47.45	15.92	161.00		.003	0.89
	n	%	*n*	%	*n*	%	χ^2	*df/n*	*p*	*V*
Gender (female)	18	32.72	6	35.29	12	31.57	0.07	1/55	.786	.04
Civil status (married/ in partnership)	28	50.91	11	64.71	17	44.74	1.87	1/55	.171	.19
Children	37	67.27	11	64.71	26	68.42	0.07	1/55	.786	.04
Education (at least university-entrance diploma)	29	52.73	9	52.94	20	52.63	0.00	1/55	.983	.00
Employment							2.43	2/55	.297	.21
Working	25	45.45	6	35.29	19	47.37				
Retired	24	43.64	10	58.82	14	36.84				
Nationality (German)	55	100.00	17	100.00	38	100.00	-	-	-	-
Alcohol Usage Disorder							1.98	1/55	.160	.19
Harmful use	9	16.36	1	5.89	8	21.05				
Addiction	46	83.64	16	94.11	30	78.94				
Hospitalization	24	43.64	-	-	24	63.16	19.05	1/55	< .001	.59
Comorbitity										
Additional Axis-I disorder(s)	29	52.73	5	29.41	24	63.16	5.37	1/55	.021	.31
Additional Axis-II-disorder(s)	3	5.45	-	-	3	7.89	1.42	1/55	.233	.16
Pharmacotherapy	16	29.09	6	35.29	10	26.32	0.04	1/43	.834	.39
Without craving-social- network	15	27.30	9	52.90	6	15.80	8.17	1/55	.004	.03
Alcohol Abstinence Self-Efficacy (KAZ-35)	74.32	21.64	91.45	9.44	66.65	21.21	85.50	1/55	<.001	1.17
Commited Action (URICA)	.23	.81	.46	.93	.13	.74	259.00	1/55	.24	.45

[a]Mann-Whitney-U-Test (*U; p*) for ordinal and continuous variables; χ^2-test (χ^2; *df/n; p) for* nominal variables

2.3 Supplementary Tables S4

Table 3

Structural aspects of the support social network compared between remitted and non-remitted patients

	Support social network									
	remitted				non-remitted					
	n	*M*	*SD*	range	*n*	*M*	*SD*	range	*z (n)*	*p*
**Network size**	17	20.64	20.59	3-46	38	23.40	18.25	4-64	-.18 (52)	.855
**Average age** (years)	17	57.53	10.23	39.22-74.58	38	46,06	10.70	24.83-68.56	-3.24 (52)	.001
**Network composition** (%)										
Total network	17				38					
Private relationships		83.47	14.74	50-100		85.87	16.56	46-100	-.96 (52)	.343
Organizational relationships		2.77	4.46	0-11		4.94	8.09	0-31	-.66 (52)	.512
Professional relationships		10.26	11.48	0-38		7.75	12.16	0-50	-1.05 (52)	.296
Others		3.50	7.25	0-25		1.44	3.97	0-17	-1.05 (52)	.296
**Network sustainability** (month)	17	356.98	228.53	72-672.00	38	254.99	225.83	12-1008	-2.70 (52)	.003
**Frequency of contact**	17	3.64	1.00	2-6	38	3.36	1.10	1-6	-1.11 (52)	.267

Annotation. All values of groups are relativized by group size.

[e] Scale from 1 to 8 [1 = daily; 2 = more than once a week; 3 = once a week; 4 = more than once a month; 5 = once a month; 6 = more than once a year; 7 = once a year; 8 = less frequent].

Table 4

Structural aspects of the craving social network compared between remitted and non-remitted patients

	Craving social network									
	remitted				non-remitted					
	n	*M*	*SD*	range	*n*	*M*	*SD*	range	*z (n)*	*p*
**Network size**	10	2.80	2.97	1-11	32	54.69	177.85	1-1006	-2.70 (42)	.007
**Average age** (years)	10	48,63	10.23	26.65-73.47	32	54,89	17.78	22.50-84.00	-1.40 (42)	.161
**Network composition** (%)										
Total network	32				10					
Private relationships		86.67	21.94	50-100		79.38	28,48	0-100	-.81 (40)	.410
Organizational relationships		.00	.00	0-0		4.86	10.31	0-29	-1.59 (40)	.111
Professional relationships		.00	.00	0-0		0.84	3.30	0-17	-.800(40)	.424
Others		13.33	21.94	0-50		14.93	27.79	0-100	-.09 (40)	.930
**Network sustainability** (month)	55	264.98	136.55	17.59-622.00	32	288.78	225.83	12.36-1008.00	-.96 (40)	.337
**Frequency of contact**	10	1.67	2.68	1-6	32	2.90	1.18	1-7	-2.26 (40)	.024

Annotation. All values of groups are relativized by group size.

[e] Scale from 1 to 8 [1 = daily; 2 = more than once a week; 3 = once a week; 4 = more than once a month; 5 = once a month; 6 = more than once a year; 7 = once a year; 8 = less frequent].
